# Characteristics of children with autism spectrum disorder in pediatric rehabilitation at a referral hospital in Peru

**DOI:** 10.17843/rpmesp.2024.411.13285

**Published:** 2024-03-26

**Authors:** Roger De la Cerna-Luna, Daniel Fernandez-Guzman, Marilia Baquerizo-Sedano, Stephanie Cabala-Olazabal, Alvaro Taype-Rondan

**Affiliations:** 1 Pediatric Rehabilitation Service, Edgardo Rebagliati Martins National Hospital, EsSalud, Lima, Peru. Pediatric Rehabilitation Service Edgardo Rebagliati Martins National Hospital EsSalud Lima Peru; 2 Human Medicine, Universidad Científica del Sur, Lima, Peru. Universidad Científica del Perú Human Medicine Universidad Científica del Sur Lima Peru; 3 Faculty of Health Sciences, University of Burgos, Burgos, Spain. University of Burgos Faculty of Health Sciences University of Burgos Burgos Spain; 4 Department of Research, Teaching and Integral Rehabilitation in Learning, National Institute of Rehabilitation, Ministry of Health, Lima, Peru. Department of Research, Teaching and Integral Rehabilitation in Learning National Institute of Rehabilitation Ministry of Health Lima Peru; 5 Research Unit for the Generation and Synthesis of Health Evidence, Vice Rectorate for Research, San Ignacio de Loyola University, Lima, Peru. San Ignacio de Loyola University Research Unit for the Generation and Synthesis of Health Evidence Vice Rectorate for Research San Ignacio de Loyola University Lima Peru

**Keywords:** Autism spectrum disorder, Child development, Physical medicine and rehabilitation, Peru

## Abstract

**Objective.:**

Autism spectrum disorder (ASD) is characterized by developmental disorders, difficulties in social interaction and communication, and restrictive and repetitive patterns of behavior. Despite its high prevalence, few studies have been conducted in rehabilitation settings. This study aimed to describe the characteristics of children with ASD from the Pediatric Rehabilitation Service of the Rebagliati Hospital (SRP-HNERM).

**Materials and methods.:**

Cross-sectional descriptive study. We reviewed the medical records of children under 14 years of age previously diagnosed with ASD from the SRP-HNERM during 2022.

**Results.:**

A total of 120 children with ASD were evaluated. The median age was 5 years. Most received regular education, but it was inclusive only for 9.8%. The mean age at diagnosis was 3.83 years. We found that 78.4% had no disability certificate and 77.5% of the participants had incomplete psychological evaluation. The median time since the last physical, occupational and speech therapy sessions was 3, 8 and 3.5 months respectively.

**Conclusion.:**

The mean age at diagnosis of ASD was older than three years, and more than 75% of the patients had neither a disability certificate nor a complete psychological evaluation. The median time since the last rehabilitation therapy sessions was three months or more. Our findings highlight the need to improve early diagnosis, inclusive education and evaluation and subsequent certification of disability, as well as to establish timely interventions.

## INTRODUCTION

Autism spectrum disorder (ASD) is characterized by alterations in child development, difficulties in social interaction, verbal and nonverbal communication, as well as restrictive and repetitive patterns of behavior, interests and activities ^1^. ASD is more frequent in males and, although it is related to genetic and environmental factors ^1^^,^^2^, its etiology is still unknown, the onset is variable, and it is usually identified in children from two years of age ^2^.

According to a recent systematic review, the overall prevalence of ASD in children would be 1% ^3^. In the United Kingdom, a similar rate has been estimated in young and older adults ^4^. No prevalence studies have been conducted in Peru, but according to the latest “Specialized Survey on Disability - ENEDIS” (2012), 3.4% of households in the country reported having a member with a limitation to relate to other people due to feelings, emotions and behaviors ^5^.

People with ASD require a legal regime that safeguards and promotes their comprehensive care and social inclusion; for this reason, since 2014, Peru has had Law No. 30150 “Law for the protection of people with ASD” ^6^. In this framework, the “National Plan for Persons with ASD 2019-2021” (2019) and the “Clinical Practice Guideline for the Diagnosis and Treatment of ASD in Children and Adolescents” (2020) have been published ^5^^,^^7^.

Early identification of children with ASD through proper diagnosis allows initiating therapeutic interventions, which should involve health professionals, family and educators ^8^. A study conducted in 2022 found that ASD was diagnosed at a later age in Peru than in other countries of the continent ^9^, which could interfere with the early initiation of therapeutic interventions.

Some studies in different countries have investigated these and other characteristics of children with ASD ^10^^-^^13^. However, despite the increasing prevalence of ASD ^2^, few studies worldwide and none in Peru have been conducted in Physical Medicine and Rehabilitation (PM&R) settings. For this reason, this study aimed to describe the characteristics of children with ASD from the Pediatric Rehabilitation Service of the Edgardo Rebagliati Martins National Hospital (SRP-HNERM) in 2022.

**KEY MESSAGES**

Motivation for the study: Despite the prevalence of ASD, research in the field of Physical Medicine and Rehabilitation is scarce in Peru.

Main findings: Of 120 children with a previous diagnosis of ASD, only 9.8% received inclusive education. The median age at diagnosis was 3.83 years. We also found that 78.4% had no disability certificate and 77.5% had incomplete psychological evaluation. The median time since the last physical, occupational, and speech therapy sessions was 3 months, 8 months, and 3.5 months, respectively.

Implications: These findings highlight the need to enhance early diagnosis, inclusive education, and evaluation and subsequent certification of disability, as well as to establish more timely interventions.

## MATERIALS AND METHODS

### Design and study population

We conducted a descriptive cross-sectional study. We included all electronic medical records of children under 14 years of age with a previous diagnosis of ASD from the SRP-HNERM between January 1 and December 31, 2022.

### Context

In Peru, a shortage of health professionals specialized in ASD has been reported ^14^ in several health systems, the largest being the Ministry of Health (MINSA), funded by the State, and the Social Health Insurance (EsSalud), funded by contributions from workers of public and private entities.

The HNERM, located in the capital of Peru, is one of the most important EsSalud national referral centers, due to its high level of specialization and resolution capacity ^15^. Therefore, all patients at the SRP-HNERM were referred from a less complex EsSalud health care center (outside Lima or the Rebagliati Health Care Network) or by interconsultation with another HNERM service.

During 2022 the SRP-HNERM had four physiatrists, fifteen physical therapists, two occupational therapists, five speech therapists and two psychologists, almost all of whom worked with children with ASD.

### Procedures

After approval by the ethics committee, we requested the list of all children with a confirmed diagnosis of ASD who were seen in medical consultation in 2022 from SRP-HNERM. Subsequently, we manually reviewed 120 electronic medical records to extract the data into a database in Microsoft Excel.

We included outpatients from the SRP-HNERM because the medical evaluation of all patients has been standardized there through a Pediatric Rehabilitation Evaluation Protocol ^16^, which allows us to better characterize this group.

### Variables

The variables of interest (general, clinical and related to assessment and management) were chosen based on the elements considered by the Pediatric Rehabilitation Assessment Protocol used in the SRP-HNERM ^16^.

We collected the following general variables: age (years), sex (female, male), origin (Lima, outside Lima), type of patient (new, continuing), regular educational modality (initial, primary, secondary), special educational modality (Early Intervention Program or PRITE, Special Basic Education Center or CEBE, Institute for Comprehensive Rehabilitation and Special Education or IRIEE), other educational modalities (early stimulation, personalized education), support from the Special Educational Needs Support and Counseling Service or SAANEE (team of professionals in charge of promoting inclusive education in regular education) ^5^ (yes, no), and reasons why the patient did not attend an educational institution.

We also collected clinical variables such as: history of prematurity (yes, no), BMI for age (underweight, normal, overweight, obese), height for age (short, adequate, tall), presence of aggressive behaviors (behaviors intended to cause harm, pain or discomfort to another person or entity ^17^, as stated by the parent or caregiver in the anamnesis) (yes, no), previous diagnosis of musculoskeletal disorder (such as hypermobility spectrum disorder, scoliosis, joint pathology, etc. ) (yes, no), degree of intellectual disability (according to psychological report included in the anamnesis, in patients with complete psychological evaluation) (mild, moderate, severe), International Classification of Diseases Tenth Edition or ICD-10 code (F84.0 or Infantile autism, F84.1 or Atypical autism, F84.2 o Rett syndrome, F84.3 or Other childhood disintegrative disorder, F84.4 or Hyperkinetic disorder with mental retardation and stereotyped movements, F84.5 or Asperger’s syndrome), age at ASD diagnosis, medical specialty that made the ASD diagnosis (PM&R, Neurology, Psychology, Psychiatry), whether they had a disability certificate (yes, no) and time elapsed without a disability certificate since ASD was diagnosed (years).

The following variables related to evaluation and management were obtained: evaluation by Neurology and Psychiatry prior to the PM&R consultation (yes, no), status of psychological evaluation (complete, incomplete), history of having received particular rehabilitation therapies (yes, no), type of rehabilitation therapy received (physical, occupational, speech), time elapsed since the last rehabilitation therapy sessions, type of child developmental delay (assessed by the Child Development Assessment Profile of the Rebagliati Hospital or REBA-PED, ^18^^,^^19^ by areas and in percentages) (simple delay, significant delay, global delay), presence of alarm signs in child development (yes, no), comorbidities, care by medical specialties at HNERM (Allergology, Cardiology, Dermatology, Endocrinology, Gastroenterology, Genetics, Hematology, Pneumology, Nephrology, Neurology, Ophthalmology, Otolaryngology, Psychiatry, Urology) and current medication (antipsychotics, antihistamines, salbutamol, methylphenidate, antiepileptics, melatonin, sertraline, others).

### Statistical analysis

We imported the data collected in the Excel spreadsheet into the R software, version 4.1.0 (R Foundation for Statistical Computing, Vienna, Austria), where we carried out all analyses and graphs. Numerical variables were presented as means and standard deviations or medians and interquartile ranges (IQR), according to the distribution of the data in normality tests. Categorical variables were presented as absolute and relative frequencies.

### Ethical aspects

This study was approved by the Institutional Research Ethics Committee of the HNERM (Ethics Qualification Certificate No. 64-CE-GHNERM-GRPR-ESSALUD-2023). All the collected information was treated with absolute confidentiality and it was exclusively used for the study.

## RESULTS

We evaluated 120 children aged 14 years or younger diagnosed with ASD attended in 2022 at the SRP-HNERM. The median age was five years (IQR: 4-7), most were male (79.2%), came from Lima (90.0%) and were continuing patients (63.3%). Regarding educational modality, most were in regular (63.54%) or special (29.17%) education. Of 61 children who received regular education, only 6 (9.84%) reported having SAANEE support (Table 1).

**Table 1:** General and clinical characteristics of children with ASD seen at the Pediatric Rehabilitation Service of the Edgardo Rebagliati Martins National Hospital, during 2022.

General and clinical characteristics		n (%)
Age ^a^		5 (4-7)
Male		95 (79.2)
From Lima		108 (90.0)
Type of patient		
	Continuing	76 (63.3)
	New	44 (36.7)
Educational modality (n=96)		
	Regular (Initial, Primary, Secondary)	61 (63.54)
	Special (CEBE, IRIEE, PRITE)	29 (30.21)
	Personalized	4 (4.17)
	Early stimulation	2 (2.08)
	SAANEE support (n=61)	6 (9.84)
	Prematurity	10 (8.33)
Weight for age		
	Low weight	11 (9.17)
	Normal	95 (79.2)
	Overweight	11 (9.17)
	Obesity	3 (2.5)
Height for age		
	High	1 (0.83)
	Low	5 (4.17)
	Adequate	114 (95.0)
	Aggressive behaviors	29 (24.2)
Previous diagnosis of musculoskeletal disorder (hypermobility spectrum disorder, scoliosis, joint condition, etc.)		15 (12.5)
Intellectual disability, according to psychological report (n=27)		
	No	2 (7.4)
	Mild	13 (48.1)
	Moderate	10 (37.0)
	Severe	2 (7.4)
ICD-10 code recorded		
	Childhood autism (F84.0)	105 (87.5)
	Asperger Syndrome (F84.5)	8 (6.67)
	Atypical autism (F84.1)	7 (5.83)
	Age at diagnosis ^b^	3.83 ± 1.67
Medical specialty that made the diagnosis		
	Neurology	51 (42.5)
	Psychiatry	32 (26.7)
	Psychology	26 (21.7)
	Physical Medicine and Rehabilitation	11 (9.17)
Has a disability certificate		26 (21.6)
Years elapsed without disability certificate since diagnosis of ASD was made (n=94) ^a^		1 (0-2)

a Median (interquartile range).

b Mean ± standard deviation.

CEBE: Center for Basic Special Education.

IRIEE: Institute for Comprehensive Rehabilitation and Special Education.

PRITE: Early Intervention Program.

SAANEE: Special Educational Needs Support and Counseling Service (team of professionals in charge of promoting inclusive education in regular education).

ICD-10: International Classification of Diseases Tenth Edition.

According to what was reported by their parents or caregivers, of 22 children who did not attend an educational institution, 15 did not attend due to their age (they were under three years old or had recently turned three years old), 4 because of aggressive behavior, 1 because of severe illness of the parent, 1 because they did not find a suitable educational institution and 1 because they were being educated at home.

The majority had normal BMI for their age (79.2%) and adequate height for their age (95.0%). Aggressive behaviors were found in 24.2% of the children and 12.5% had a previous diagnosis of musculoskeletal disorder. Of 27 who had a complete psychological evaluation, most had mild (48.1%) or moderate (37.0%) intellectual disability. The most frequent ICD-10s recorded in the electronic medical record were F84.0 or Childhood Autism (87.5%) and F84.5 or Asperger’s Syndrome (6.67%) (Table 1).

The mean age at ASD diagnosis was 3.83 years. Neurology was the specialty that most frequently made the diagnosis of ASD (42.5%), followed by Psychiatry (26.7%). We also found that 78.4% did not have a disability certificate. The median time without a disability certificate since the diagnosis of ASD was made was 1 year (IQR: 0-2) (Table 1).

We found that 60.0% had been previously by the Neurology service and 64.2% by the Psychiatry service. Psychological evaluation was incomplete in 77.5%. Regarding the treatment, 26.7% had received private therapies (paid for by their parents or caregivers), 49.2% received physical therapy, 95.0% received occupational therapy and 90.0% received speech therapy. The median time since the last rehabilitation therapy session varied by type: for physical therapy it was 3 months (IQR: 1-6), for occupational therapy it was 8 months (IQR: 1-24) and for speech therapy it was 3.5 months (IQR: 1-20) (Table 2).

**Table 2:** Previous evaluations, rehabilitation therapies and developmental delay of children with ASD from the Pediatric Rehabilitation Service of the Edgardo Rebagliati Martins National Hospital, during 2022.

Preliminary evaluations, rehabilitation therapies and developmental delay		n (%)
	Previous evaluation by Neurology	72 (60.0)
	Previous evaluation by Psychiatry	77 (64.2)
	Complete psychological evaluation	27 (22.5)
	Received private rehabilitation therapies	32 (26.7)
	Received physical therapy	59 (49.2)
	Received occupational therapy	114 (95.0)
	Received speech therapy	108 (90.0)
Time in months since last therapy session ^a^		
	Physical therapy (n=40)	3.0 (1.0-6.0)
	Occupational therapy (n=53)	8.0 (1.0-24.0)
	Speech therapy (n=56)	3.5 (1.0-20.0)
Childhood developmental delay (under 5 years; n=51)		
	No	2 (3.9)
	Simple (< 25% in one or more areas)	2 (3.9)
	Significant (≥ 25% in an area)	4 (7.8)
	Global (≥ 25% in two areas or more)	43 (84.3)
	Presence of warning signs (under 5 years; n=51)	50 (98.0)

a Median (interquartile range).

**Figure1. Percentage:**
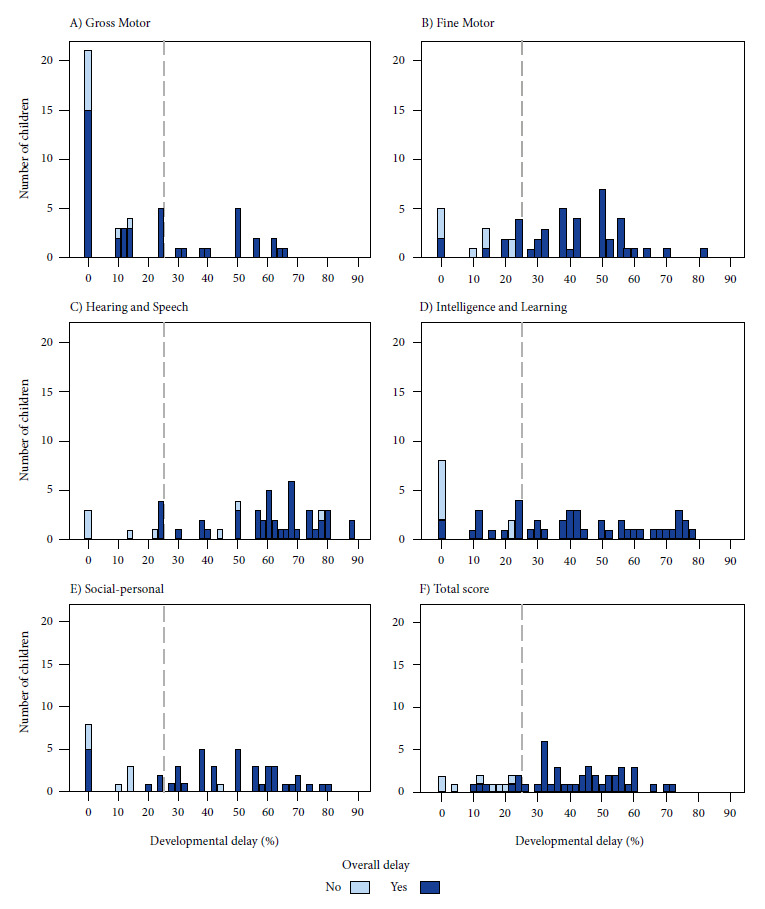
of developmental delay, by area, in children under five years of age with ASD from the Pediatric Rehabilitation Service of the Edgardo Rebagliati Martins National Hospital, during 2022 (n=51).

Regarding the evaluation of child development by REBA-PED in those under five years of age, 53.5% had global developmental delay (≥ 25% in two areas or more), 19.5% significant delay (≥ 25% in one area) and 12.4% simple delay (< 25% in one or more areas). In addition, 70.8% had some sign of alarm (Table 2). For a better visualization of the distribution of developmental delay, a histogram graph is presented for each area of child development and differentiating those with global developmental delay and those without global developmental delay (Figure 1).

On the other hand, 71.8% did not have any comorbidity, and among those who did, the most frequent was attention deficit hyperactivity disorder (ADHD) (12.5%), followed by neurological malformations (9.2%), epilepsy (4.2%), insomnia (3.3%), anxiety (1.7%) and depression (1.7%). The medical specialties with the highest number of visits were Psychiatry (33.3%), Pneumology (13.3%), Neurology (12.5%), Genetics (10.0%) and Gastroenterology (10.0%). The most frequently used drugs were antipsychotics (35.8%), mainly risperidone, antihistamines (10.8%), salbutamol (10.8%), methylphenidate (6.7%) and antiepileptics (5%) (Table 3).

**Table 3:** Comorbidities, visits to medical specialties and current medication of children with ASD from the Pediatric Rehabilitation Service of the Edgardo Rebagliati Martins National Hospital, during 2022.

Characteristics		n (%)
Comorbidities		
	ADHD	15 (12.5)
	Neurological malformations	11 (9.2)
	Epilepsy	5 (4.2)
	Insomnia	4 (3.3)
	Anxiety	2 (1.7)
	Depression	2 (1.7)
	None	86 (71.8)
Visits to medical specialties		
	Psychiatry	40 (33.3)
	Pneumology	16 (13.3)
	Neurology	15 (12.5)
	Genetics	12 (10.0)
	Gastroenterology	12 (10.0)
	Endocrinology	11 (9.2)
	Dermatology	9 (7.5)
	Urology	6 (5.0)
	Cardiology	5 (4.2)
	Ophthalmology	5 (4.2)
	Nephrology	5 (4.2)
	Otorhinolaryngology	4 (3.3)
	Hematology	4 (3.3)
	Allergology	4 (3.3)
	None	37 (30.8)
Current medication		
	Antipsychotics	43 (35.8)
	Risperidone	19 (15.8)
	Periciazine	5 (4.2)
	Clobazam	5 (4.2)
	Chlorpromazine	4 (3.3)
	Valproate	3 (2.5)
	Haloperidol	3 (2.5)
	Aripiprazole	2 (1.7)
	Topiramate	2 (1.7)
	Antihistaminic	13 (10.8)
	Salbutamol	13 (10.8)
	Methylphenidate	8 (6.7)
	Antiepileptics	6 (5.0)
	Levetiracetam	4 (3.3)
	Carbamazepine	2 (1.7)
	Melatonin	4 (3.3)
	Sertraline	3 (2.5)
	Other	21 (17.5)

ADHD: attention deficit/hyperactivity disorder.

## DISCUSSION

We evaluated 120 children with a previous diagnosis of ASD. The majority were male. The median age at diagnosis was 3.83 years. The most frequent ICD-10 was F84.0 or Infantile Autism. Most were diagnosed by the Neurology service, and were receiving regular education. Less than a quarter of the patients presented aggressive behaviors. Most did not have a disability certificate or a complete psychological evaluation. Most had no comorbidities, presented global developmental delay and had some signs of child developmental alarm. The median time since the last rehabilitation therapy sessions ranged from three to eight months, depending on the type. Antipsychotics and methylphenidate were among the most frequently used drugs.

We found more boys than girls with ASD, in a ratio of 3.8 to 1, this coincides with previous reports (higher prevalence in males than females in a ratio of 3 to 1) ^8^, as well as with the Registry of the National Council for the Integration of Persons with Disabilities (CONADIS) of Peru, which reported that as of 2018, 80.9% of the patients registered with ASD were male ^5^. Whether this is due to biases in diagnosis or physiological and social causes that allow women to have better adaptive/compensatory behaviors is still under research ^8^.

The mean age at diagnosis of ASD was 3.83 years (46 months), close to the global estimate of 43.2 months (range: 30.9 - 74.7 months) in children under ten years of age ^20^. However, this finding suggests that we could be facing late diagnosis when compared to other countries such as Argentina or Spain (mean of 3.30 years), which could be related to extended waiting time for medical and psychological care ^9^.

Diagnoses are recorded using ICD-10 codes in EsSalud health care centers. Since 2013, the Diagnostic and Statistical Manual of Mental Disorders Fifth Edition (DSM-5) has been considered the reference for the diagnosis of ASD worldwide ^8^. The DSM-5 for ASD does not consider categories but levels of support (level 1: requires support, level 2: requires notable support, level 3: requires very notable support) ^7^. Recording the ASD support level could be useful for designing intervention programs to address specific needs, such as educational needs.

We found that 21.7% children were diagnosed with ASD by the psychology service. In Peru, MINSA stated in 2022 that the diagnosis should be made by physicians, ideally pediatric subspecialists, with the contribution of other professionals ^21^. Clinical practice guidelines on ASD usually recommend the participation of a multidisciplinary team in the diagnostic process ^22^^,^^23^. This process, which should take a maximum of three to six months, can take up to a year in high-income countries such as Canada, so it has been proposed that trained non-medical health professionals can make the diagnosis of ASD in less complex cases ^23^. This strategy could be useful to expand ASD diagnostic capacity in Peru.

The educational distribution was similar to that reported by the Peruvian Ministry of Education, with a predominance of regular education ^5^; less than 10% reported having support from SAANEE (inclusive education). A study conducted in Lima in 2022 found that there was a strong tendency to exclude children with ASD from regular education institutions, since more than 70% of the parents surveyed reported that their children were rejected from at least one school, and more than 50% reported that they had passed through more than one school ^24^. In addition, there is emerging evidence which suggests that adolescents with ASD are less likely to receive higher education than their peers without ASD ^25^.

One out of four children presented aggressive behaviors, less than that reported in the literature (35-50%) ^26^. Aggressive behaviors in children with ASD affect their education, causing non-compliance and dropping out of school, decreasing the effectiveness of rehabilitation interventions, affecting interpersonal relationships, causing social isolation and generating stress in parents, being one of the main reasons for seeking pharmacological treatment for the child ^26^.

More than 77% of the children had not completed their psychological evaluation, which may be related to the shortage of psychologists in the Peruvian health system, particularly at the first level of care and outside the capital (Lima) ^27^, and to the fact that the SRP-HNERM had only two female psychologists in 2022. Psychological assessment is fundamental when dealing with child development disorders, in order to describe functioning of several domains, particularly in everyday situations, as well as to quantify the child’s general level of cognitive development and to determine the presence or absence of intellectual disability ^28^.

Most participants did not have a disability certificate, a document that certifies the disability status of a person in Peru and allows his or her registration in CONADIS ^22^. In Peru, not all people with ASD have a disability certificate, especially in cities outside Lima, due to a lack of trained medical personnel to perform the necessary evaluation, difficulties in accessing health services, lack of knowledge and also because it is not mandatory ^22^. This situation could be improved in Peru by making the disability certificate mandatory and creating a national registry of people with ASD.

We found higher rates of developmental delay in the areas of Hearing and Language, Fine Motor and Social Personal. These findings are similar to those of a study that evaluated child development in children under five years of age at the SRP-HNERM in 2022 ^19^. Also, less than half received physical therapy, which may be related to the low frequency of developmental delay in the Gross Motor area, compared to other areas, as well as associated musculoskeletal disorders. This confirms the importance of having more occupational therapists, speech therapists and learning specialists in the SRP-HNERM.

The median wait time for occupational therapy was eight months, longer than for physical therapy and speech therapy, possibly because SRP-HNERM had only two occupational therapists during 2022. A 2016 study in the United States reported that in Tennessee, wait times for evaluations and interventions for children with ASD could be as long as six to twelve months ^29^.

The World Health Organization (WHO) published in 2023 the Rehabilitation Interventions Package for Neurodevelopmental Disorders, which details intervention for people with ASD, by physiatrists, rehabilitation therapists, psychologists, among other health professionals involved, for the assessment and management of cognitive functions, sleep functions, behavioral problems, perceptual functions, language, speech and communication functions, sexual functions and intimate relationships, movement functions, exercise tolerance functions, activities of daily living, interpersonal relationships and interactions, education, work and employment, participation in social and community life, self-care, caregiver and family support, and mental health ^30^.

Most participants did not have associated comorbidities. We believe that this could be due to underreporting, since in 2021 a systematic literature review concluded that comorbidities are highly prevalent in ASD ^31^. ADHD was the most frequent comorbidity in the children with ASD, coinciding with previous literature, which shows that the prevalence rate in this population can reach 86% ^31^. The presence of both conditions is associated with greater severity of ASD and a significantly higher risk of a third comorbidity, especially anxiety or depression ^32^. Comorbidities can pose a diagnostic and therapeutic challenge in children with ASD, requiring personalized and tailored treatment and support approaches ^31^.

Risperidone was the most prescribed medication, which is approved for use in children with ASD in the United States to treat irritability and associated aggressive behaviors ^33^. A systematic review and meta-analysis of risperidone use in children with ASD suggested that it may be effective in the treatment of lethargy and inappropriate speech ^33^. We found that methylphenidate was another commonly used medication, usually prescribed to treat the cardinal symptoms of ADHD (inattention, impulsivity, hyperactivity) ^34^. A systematic review and meta-analysis on the use of methylphenidate in children with ASD supported its efficacy and tolerability for the treatment of ADHD symptoms in this population ^34^.

This study has some limitations that should be considered in order to correctly interpret the results: 1) Since it was a retrospective study that reviewed electronic medical records, it is possible that some data may have been erroneously recorded by the physicians in charge of the consultations in the SRP-HNERM. 2) Other relevant variables could not be collected because they were not systematically recorded in the electronic medical records (such as signs and symptoms of ASD or the degree of disability at certification). 3) The study was conducted in a referral hospital in Lima, Peru, so the results may not be representative of other centers. 4) The diagnosis of ASD was made by different specialists, and we do not have accurate information on the methods they used to make this diagnosis.

However, this is one of the first Peruvian studies that has evaluated, in depth, the characteristics of children with ASD receiving care in Pediatric Rehabilitation. Furthermore, to our knowledge, this is the first study to address this issue after the COVID-19 pandemic in Peru, which provides relevant information to understand the needs of this population and to make proposals for improvement.

In summary, this study provides an in-depth look at the characteristics of children with ASD from the SRP-HNERM in Peru during 2022. The mean age of ASD diagnosis was higher than three years, and more than 75% of patients had neither a disability certificate nor a complete psychological evaluation. The education received by less than 10% of those receiving regular education could be considered as inclusive. Some did not receive rehabilitation therapies and the median time since the last session ranged from three to eight months, depending on the type. These findings highlight the urgent need to promote early diagnosis, inclusive education and the evaluation and subsequent certification of disability in children with ASD, as well as to establish more timely rehabilitation interventions in the Peruvian context.
