# Supplementary material for: Characteristics of children with autism spectrum disorder in pediatric rehabilitation at a referral hospital in Peru
Source: Rev Peru Med Exp Salud Publica. 2024 Mar 26;41(1):19–27. doi: 10.17843/rpmesp.2024.411.13285 (PMC11149770; doi:10.17843/rpmesp.2024.411.13285)
Supplement: Translation

# Translation: Características de niños con trastorno del espectro autista en rehabilitación pediátrica de un hospital de referencia en Perú

**Authors:** Roger De la Cerna-Luna, Daniel Fernandez-Guzman, Marilia Baquerizo-Sedano, Stephanie Cabala-Olazabal, Alvaro Taype-Rondan

**Affiliations:** 1 Servicio de Rehabilitación Pediátrica, Hospital Nacional Edgardo Rebagliati Martins, EsSalud, Lima, Perú.; 2 Carrera de Medicina Humana, Universidad Científica del Sur, Lima, Perú.; 3 Facultad de Ciencias de la Salud, Universidad de Burgos, Burgos, España.; 4 Departamento de Investigación, Docencia y Rehabilitación Integral en el Aprendizaje, Instituto Nacional de Rehabilitación, Ministerio de Salud, Lima, Perú.; 5 Unidad de Investigación para la Generación y Síntesis de Evidencia en Salud, Vicerrectorado de Investigación, Universidad San Ignacio de Loyola, Lima, Perú.

**Keywords:** Trastorno del Espectro Autista, Desarrollo Infantil, Medicina Física y Rehabilitación, Perú

## Abstract

**Objetivo.:**

El trastorno del espectro autista (TEA) se caracteriza por alteraciones en el desarrollo, dificultades de interacción social y comunicación, y patrones restrictivos y repetitivos de conducta. A pesar de su alta prevalencia, pocos estudios se han realizado en ambientes de rehabilitación. El objetivo del estudio fue describir las características de los niños con TEA atendidos en el Servicio de Rehabilitación Pediátrica del Hospital Rebagliati (SRP-HNERM).

**Materiales y métodos.:**

Estudio descriptivo transversal. Se revisaron las historias clínicas de niños menores de 14 años con diagnóstico previo de TEA atendidos en el SRP-HNERM durante el 2022.

**Resultados.:**

Se evaluaron a 120 niños con TEA. La mediana de edad fue de cinco años. La mayoría recibía educación regular, pero solo para el 9,8% fue inclusiva. La media de edad de diagnóstico fue de 3,83 años. El 78,4% no tenía certificado de discapacidad y el 77,5% tenía evaluación psicológica incompleta. La mediana del tiempo transcurrido desde las últimas sesiones de terapia física, ocupacional y de lenguaje fue de 3, 8 y 3,5 meses respectivamente.

**Conclusión.:**

La media de edad de diagnóstico de TEA fue mayor de tres años y más del 75% de los pacientes no contaban con un certificado de discapacidad ni con una evaluación psicológica completa. La mediana del tiempo desde las últimas sesiones de terapias de rehabilitación fue de tres meses o más. Estos hallazgos resaltan la necesidad de potenciar el diagnóstico temprano, la educación inclusiva y la evaluación y posterior certificación de la discapacidad, así como de establecer intervenciones más oportunas.

## INTRODUCCIÓN

El trastorno del espectro autista (TEA) se caracteriza por alteraciones en el desarrollo infantil, dificultades en la interacción social y en la comunicación verbal y no verbal, y patrones restrictivos y repetitivos de conducta, intereses y actividades ^1^. El TEA tiene mayor prevalencia en el sexo masculino y, si bien guarda relación con factores genéticos y ambientales ^1^^,^^2^, su etiología es aún desconocida, el inicio es variable, y suele identificarse en niños a partir de los dos años ^2^.

Según una revisión sistemática reciente, la prevalencia global del TEA en niños sería de 1% ^3^. En el Reino Unido se ha estimado un porcentaje similar en adultos jóvenes y mayores ^4^. En el Perú no se han realizado estudios de prevalencia, pero, según la última «Encuesta Especializada sobre Discapacidad - ENEDIS» (2012), el 3,4% de hogares en el país refirió tener un miembro con limitación para relacionarse con los demás por sus sentimientos, emociones y conductas ^5^.

Las personas con TEA requieren un régimen legal que resguarde y promueva su atención integral e inclusión social; por tal motivo, desde el 2014 existe en el Perú la Ley N.º 30150 «Ley de protección de las personas con TEA» ^6^. En ese marco, se han publicado el «Plan Nacional para las Personas con TEA 2019-2021» (2019) y la «Guía de Práctica Clínica para el Diagnóstico y Tratamiento del TEA en Niños y Adolescentes» (2020) ^5^^,^^7^.

La identificación temprana de los niños con TEA mediante un diagnóstico adecuado permite iniciar intervenciones terapéuticas, que deben involucrar a los profesionales de la salud, la familia y los educadores ^8^. Un estudio realizado el 2022 halló una edad de diagnóstico de TEA en el Perú más tardía que en otros países del continente ^9^, lo que podría interferir con el inicio precoz de las intervenciones terapéuticas.

Algunos estudios en distintos países han investigado estas y otras características de niños con TEA ^10^^-^^13^. Sin embargo, a pesar de que el TEA es cada vez más prevalente ^2^, pocos estudios a nivel mundial y ninguno en el Perú han sido realizados en ambientes de Medicina Física y Rehabilitación (MFyR). Por tal motivo, se realizó el presente estudio con el objetivo de describir las características de los niños con TEA atendidos durante el 2022 en el Servicio de Rehabilitación Pediátrica del Hospital Nacional Edgardo Rebagliati Martins (SRP-HNERM).

**MENSAJE CLAVE**

Motivación para realizar el estudio: A pesar de la prevalencia del trastorno del espectro autista (TEA), la investigación en el campo de la Medicina Física y Rehabilitación es escasa en Perú.

Principales hallazgos: De 120 niños con diagnóstico previo de TEA evaluados, solo el 9,8% recibió educación inclusiva. La media de edad de diagnóstico fue de 3,83 años. El 78,4% no tenía certificado de discapacidad y el 77,5% tenía evaluación psicológica incompleta. La mediana del tiempo transcurrido desde las últimas sesiones de terapia física, ocupacional y de lenguaje fue de 3 meses, 8 meses y 3,5 meses, respectivamente.

Implicancias: Estos hallazgos resaltan la necesidad de potenciar el diagnóstico temprano, la educación inclusiva y la evaluación y posterior certificación de la discapacidad, así como de establecer intervenciones más oportunas.

## MATERIALES Y MÉTODOS

### Diseño y población de estudio

Realizamos un estudio descriptivo de tipo transversal. Incluimos a todas las historias clínicas electrónicas de los niños menores de 14 años con diagnóstico previo de TEA atendidos en el SRP-HNERM entre el 1 de enero y el 31 de diciembre del 2022.

### Contexto

En el Perú se ha reportado escasez de profesionales de la salud especializados en TEA ^14^, que trabajan en diversos sistemas de salud, siendo los más grandes el del Ministerio de Salud (MINSA), financiado por el Estado, y el del Seguro Social de Salud (EsSalud), financiado por aportes de los trabajadores de entidades públicas y privadas.

El HNERM, ubicado en la capital del Perú, es uno de los más importantes centros de referencia nacional de EsSalud, debido a su alto nivel de especialización y capacidad resolutiva ^15^. Por ello, todos los pacientes en el SRP-HNERM fueron atendidos por referencia de algún centro asistencial de EsSalud de menor complejidad (fuera de Lima o de la Red Prestacional Rebagliati) o por interconsulta de algún otro servicio del HNERM.

Durante el 2022 el SPR-HNERM contó con cuatro fisiatras, quince terapeutas físicos, dos terapeutas ocupacionales, cinco terapeutas de lenguaje y dos psicólogas, de los cuales casi todos atendieron a niños con TEA.

### Procedimientos

Tras la aprobación por el comité de ética, solicitamos al SRP-HNERM la lista de todos los niños con diagnóstico confirmado de TEA que fueron atendidos en consulta médica en el año 2022. Posteriormente, revisamos manualmente 120 historias clínicas electrónicas para extraer los datos de interés, a una base de datos en Microsoft Excel.

Consideramos a los pacientes atendidos en consulta médica del SRP-HNERM debido a que en dicho lugar se ha estandarizado la evaluación médica de todos los pacientes a través de un Protocolo de Evaluación de Rehabilitación Pediátrica ^16^, lo que permite caracterizar mejor a este grupo.

### Variables

La elección de las variables de interés (generales, clínicas y relacionadas con la evaluación y el manejo) se fundamentó en los elementos considerados por el Protocolo de Evaluación de Rehabilitación Pediátrica que se usa en el SRP-HNERM ^16^.

Se recopilaron las siguientes variables generales: edad (en años), sexo (femenino, masculino), procedencia (Lima, fuera de Lima), tipo de paciente (nuevo, continuador), modalidad educativa regular (inicial, primaria, secundaria), modalidad educativa especial (Programa de Intervención Temprana o PRITE, Centro de Educación Básica Especial o CEBE, Instituto de Rehabilitación Integral y Educación Especial o IRIEE), otras modalidades educativas (estimulación temprana, educación personalizada), apoyo del Servicio de Apoyo y Asesoramiento a las Necesidades Educativas Especiales o SAANEE (equipo de profesionales encargados de promover la educación inclusiva en la educación regular) ^5^ (sí, no), y motivos por los cuales el paciente no asistía a una institución educativa.

Se recolectaron también variables clínicas como: antecedente de prematuridad (sí, no), IMC para la edad (bajo peso, normal, sobrepeso, obesidad), talla para la edad (baja, adecuada, alta), presencia de conductas agresivas (comportamientos que tengan la intención de causar daño, dolor o malestar a otra persona o entidad ^17^, según lo manifestado por el padre o cuidador en la anamnesis) (sí, no), diagnóstico previo de trastorno musculoesquelético (como trastorno del espectro de hipermovilidad, escoliosis, patología articular, etc.) (sí, no), grado de discapacidad intelectual (según informe psicológico incluido en la anamnesis, en pacientes con evaluación psicológica completa) (leve, moderada, severa), código de la Clasificación Internacional de Enfermedades Décima Edición o CIE-10 (F84.0 o Autismo infantil, F84.1 o Autismo atípico, F84.2 o Síndrome de Rett, F84.3 u Otro trastorno desintegrativo de la infancia, F84.4 o Trastorno hipercinético con retraso mental y movimientos estereotipados, F84.5 o Síndrome de Asperger), edad de diagnóstico de TEA, especialidad que realizó el diagnóstico de TEA (MFyR, Neurología, Psicología, Psiquiatría), si tiene certificado de discapacidad (sí, no) y tiempo transcurrido sin certificado de discapacidad desde que se realizó el diagnóstico de TEA (en años).

Con respecto a las variables relacionadas a la evaluación y el manejo, registramos: evaluación por Neurología y Psiquiatría previa a la consulta de MFyR (sí, no), estado de la evaluación psicológica (completa, incompleta), antecedente de haber recibido terapias de rehabilitación particulares (sí, no), tipo de terapia de rehabilitación recibida (física, ocupacional, de lenguaje), tiempo transcurrido desde las últimas sesiones de terapia de rehabilitación, tipo de retraso del desarrollo infantil (evaluado mediante el Perfil de Evaluación del Desarrollo Infantil del Hospital Rebagliati o REBA-PED,^18^^,^^19^ por áreas y en porcentajes) (retraso simple, retraso significativo, retraso global), presencia de signos de alarma en el desarrollo infantil (sí, no), comorbilidades, atenciones por especialidades médicas en el HNERM (Alergología, Cardiología, Dermatología, Endocrinología, Gastroenterología, Genética, Hematología, Neumología, Nefrología, Neurología, Oftalmología, Otorrinolaringología, Psiquiatría, Urología) y medicación actual (antipsicóticos, antihistamínicos, salbutamol, metilfenidato, antiepilépticos, melatonina, sertralina, otros).

### Análisis estadístico

Importamos los datos recolectados de la hoja de cálculo de Excel al software R, versión 4.1.0 (R Foundation for Statistical Computing, Viena, Austria), donde ejecutamos todos los análisis y gráficos. Las variables numéricas fueron presentadas como medias y desviaciones estándar o medianas y rangos intercuartílicos (RIC), de acuerdo con la distribución de los datos en las pruebas de normalidad. Las variables categóricas fueron presentadas con frecuencias absolutas y relativas.

### Aspectos éticos

El presente estudio fue aprobado por el Comité Institucional de Ética en Investigación del HNERM (Certificado de Calificación Ética N° 64-CE-GHNERM-GRPR-ESSALUD-2023). Toda la información recolectada permaneció en absoluta confidencialidad y su uso fue exclusivo para el estudio.

## RESULTADOS

Evaluamos a 120 niños con una edad menor o igual a 14 años con diagnóstico de TEA atendidos el 2022 en el SRP-HNERM. La mediana de edad fue de cinco años (RIC 4-7), la mayoría fue de sexo masculino (79,2%), provenían de Lima (90,0%) y eran pacientes continuadores (63,3%). Respecto a la modalidad educativa, la mayoría se encontraba en regular (63,54%) o especial (29,17%). De 61 que recibían educación regular, solo 6 (9,84%) refirieron contar con apoyo del SAANEE (Tabla 1).

**Tabla 1:** Características generales y clínicas de niños con TEA atendidos en el Servicio de Rehabilitación Pediátrica del Hospital Nacional Edgardo Rebagliati Martins, durante el 2022.

Características generales y clínicas		n (%)
Edad ^a^		5 (4-7)
Sexo masculino		95 (79,2)
Procedente de Lima		108 (90,0)
Tipo de paciente		
	Continuador	76 (63,3)
	Nuevo	44 (36,7)
Modalidad educativa (n=96)		
	Regular (Inicial, Primaria, Secundaria)	61 (63,54)
	Especial (CEBE, IRIEE, PRITE)	29 (30,21)
	Personalizada	4 (4,17)
	Estimulación temprana	2 (2,08)
	Apoyo del SAANEE (n=61)	6 (9,84)
	Prematuridad	10 (8,33)
Peso para la edad		
	Bajo peso	11 (9,17)
	Normal	95 (79,2)
	Sobrepeso	11 (9,17)
	Obesidad	3 (2,5)
Talla para la edad		
	Alta	1 (0,83)
	Baja	5 (4,17)
	Adecuada	114 (95,0)
	Conductas agresivas	29 (24,2)
Diagnóstico previo de trastorno musculoesquelético (trastorno del espectro de hipermovilidad, escoliosis, patología articular, etc.)		15 (12,5)
Discapacidad intelectual, según informe psicológico (n=27)		
	No	2 (7,4)
	Leve	13 (48,1)
	Moderada	10 (37,0)
	Severa	2 (7,4)
Código CIE-10 registrado		
	Autismo infantil (F84.0)	105 (87,5)
	Síndrome de Asperger (F84.5)	8 (6,67)
	Autismo atípico (F84.1)	7 (5,83)
Edad de diagnóstico ^b^		3,83 ± 1,67
Especialidad que realizó el diagnóstico		
	Neurología	51 (42,5)
	Psiquiatría	32 (26,7)
	Psicología	26 (21,7)
	Medicina Física y Rehabilitación	11 (9,17)
Tiene certificado de discapacidad		26 (21,6)
Años transcurridos sin certificado de discapacidad desde que se realizó el diagnóstico de TEA (n=94) ^a^		1 (0-2)

a Mediana (rango intercuartílico).

b Media ± desviación estándar.

CEBE: Centro de Educación Básica Especial.

IRIEE: Instituto de Rehabilitación Integral y Educación Especial.

PRITE: Programa de Intervención Temprana.

SAANEE: Servicio de Apoyo y Asesoramiento a las Necesidades Educativas Especiales (equipo de profesionales encargados de promover la educación inclusiva en la educación regular).

CIE-10: Clasificación Internacional de Enfermedades Décima Edición.

Según lo referido por sus padres o cuidadores, de 22 niños que no acudían a una institución educativa, 15 fue debido a su edad (eran menores de tres años o hace poco habían cumplido los tres años), 4 por conductas agresivas, 1 por enfermedad severa del padre, 1 porque no encontraron una institución educativa adecuada y 1 porque recibía educación en casa.

La mayoría tuvo IMC normal para la edad (79,2%) y talla adecuada para la edad (95,0%). El 24,2% presentó conductas agresivas y el 12,5% diagnóstico previo de trastorno musculoesquelético. De 27 que contaban con evaluación psicológica completa, la mayoría tuvo discapacidad intelectual leve (48,1%) o moderada (37,0%). Los CIE-10 más comunes registrados en la historia clínica electrónica fueron F84.0 o Autismo infantil (87,5%) y F84.5 o Síndrome de Asperger (6,67%) (Tabla 1).

La media de edad de diagnóstico de TEA fue de 3,83 años. Neurología fue la especialidad que más frecuentemente realizó el diagnóstico de TEA (42,5%), seguida por Psiquiatría (26,7%). El 78,4% no tenía certificado de discapacidad. La mediana del tiempo sin certificado de discapacidad desde que se realizó el diagnóstico de TEA fue de 1 año (RIC 0-2) (Tabla 1).

El 60,0% tuvo una evaluación previa por Neurología y el 64,2% por Psiquiatría. El 77,5% tenía evaluación psicológica incompleta. En cuanto al tratamiento recibido, el 26,7% había recibido terapias particulares (pagadas por sus padres o cuidadores), el 49,2% recibió terapia física, el 95,0% terapia ocupacional y el 90,0% terapia de lenguaje. La mediana del tiempo desde la última sesión de terapia de rehabilitación varió según el tipo: para terapia física fue de 3 meses (RIC 1-6), para terapia ocupacional fue de 8 meses (RIC 1-24) y para terapia de lenguaje fue de 3,5 meses (RIC 1-20) (Tabla 2).

**Tabla 2:** Evaluaciones previas, terapias de rehabilitación y retraso del desarrollo de niños con TEA atendidos en el Servicio de Rehabilitación Pediátrica del Hospital Nacional Edgardo Rebagliati Martins, durante el 2022.

Evaluaciones previas, terapias de rehabilitación y retraso del desarrollo		n (%)
	Evaluación previa por Neurología	72 (60,0)
	Evaluación previa por Psiquiatría	77 (64,2)
	Evaluación psicológica completa	27 (22,5)
	Recibió terapias de rehabilitación particulares	32 (26,7)
	Recibió terapia física	59 (49,2)
	Recibió terapia ocupacional	114 (95,0)
	Recibió terapia de lenguaje	108 (90,0)
Tiempo en meses desde la última sesión de terapia ^a^		
	Terapia física (n=40)	3,0 (1,0-6,0)
	Terapia ocupacional (n=53)	8,0 (1,0-24,0)
	Terapia del lenguaje (n=56)	3,5 (1,0-20,0)
Retraso del desarrollo infantil (menores de 5 años; n=51)		
	No	2 (3,9)
	Simple (< 25% en una o más áreas)	2 (3,9)
	Significativo (≥ 25% en un área)	4 (7,8)
	Global (≥ 25% en dos áreas o más)	43 (84,3)
	Presencia de signos de alarma (menores de 5 años; n=51)	50 (98,0)

a Mediana (rango intercuartílico).

En la evaluación del desarrollo infantil a través del REBA-PED en aquellos menores de cinco años, el 53,5% presentó retraso global del desarrollo (≥ 25% en dos áreas o más), el 19,5% retraso significativo (≥ 25% en un área) y el 12,4% retraso simple (< 25% en una o más áreas). Además, el 70,8% tenía algún signo de alarma (Tabla 2). Para una mejor visualización de la distribución del retraso del desarrollo se presenta un gráfico de histograma por cada área del desarrollo infantil evaluada y diferenciando a aquellos con retraso global del desarrollo y sin retraso global del desarrollo (Figura 1).

**Figura 1:** Porcentaje de retraso del desarrollo, por áreas, en niños menores de cinco años con TEA atendidos en el Servicio de Rehabilitación Pediá-trica del Hospital Nacional Edgardo Rebagliati Martins, durante el 2022 (n=51).

Por otro lado, el 71,8% no tenía ninguna comorbilidad, y en aquellos que tuvieron la más frecuente fue trastorno por déficit de atención e hiperactividad (TDAH) (12,5%), seguida de malformaciones neurológicas (9,2%), epilepsia (4,2%), insomnio (3,3%), ansiedad (1,7%) y depresión (1,7%). Las especialidades médicas que registraban más atenciones fueron Psiquiatría (33,3%), Neumología (13,3%), Neurología (12,5%), Genética (10,0%) y Gastroenterología (10,0%). Los fármacos más frecuentemente usados fueron los antipsicóticos (35,8%), principalmente la risperidona, los antihistamínicos (10,8%), el salbutamol (10,8%), el metilfenidato (6,7%) y los antiepilépticos (5%) (Tabla 3).

**Tabla 3:** Comorbilidades, atenciones por especialidades médicas y medicación actual de niños con TEA atendidos en el Servicio de Rehabilitación Pediátrica del Hospital Nacional Edgardo Rebagliati Martins, durante el 2022.

Características		n (%)
Comorbilidades		
	TDAH	15 (12,5)
	Malformaciones neurológicas	11 (9,2)
	Epilepsia	5 (4,2)
	Insomnio	4 (3,3)
	Ansiedad	2 (1,7)
	Depresión	2 (1,7)
	Ninguna	86 (71,8)
Atenciones por especialidades médicas		
	Psiquiatría	40 (33,3)
	Neumología	16 (13,3)
	Neurología	15 (12,5)
	Genética	12 (10,0)
	Gastroenterología	12 (10,0)
	Endocrinología	11 (9,2)
	Dermatología	9 (7,5)
	Urología	6 (5,0)
	Cardiología	5 (4,2)
	Oftalmología	5 (4,2)
	Nefrología	5 (4,2)
	Otorrinolaringología	4 (3,3)
	Hematología	4 (3,3)
	Alergología	4 (3,3)
	Ninguna	37 (30,8)
Medicación actual		
	Antipsicóticos	43 (35,8)
	Risperidona	19 (15,8)
	Periciazina	5 (4,2)
	Clobazam	5 (4,2)
	Clorpromazina	4 (3,3)
	Valproato	3 (2,5)
	Haloperidol	3 (2,5)
	Aripiprazol	2 (1,7)
	Topiramato	2 (1,7)
	Antihistamínicos	13 (10,8)
	Salbutamol	13 (10,8)
	Metilfenidato	8 (6,7)
	Antiepilépticos	6 (5,0)
	Levetiracetam	4 (3,3)
	Carbamazepina	2 (1,7)
	Melatonina	4 (3,3)
	Sertralina	3 (2,5)
	Otros	21 (17,5)

TDAH: trastorno por déficit de atención e hiperactividad.

## DISCUSIÓN

Se evaluaron a 120 niños con diagnóstico previo de TEA. La mayoría era de sexo masculino. La media de edad de diagnóstico fue de 3,83 años. El CIE-10 más comúnmente registrado fue F84.0 o Autismo infantil. La mayoría fue diagnosticada por Neurología, y recibía educación regular. Menos de un cuarto de pacientes presentó conductas agresivas. La mayoría no contaba con un certificado de discapacidad ni con una evaluación psicológica completa. La mayoría no tenía comorbilidades, presentó retraso global del desarrollo y tenía algún signo de alarma del desarrollo infantil. La mediana del tiempo desde las últimas sesiones de terapias de rehabilitación varió entre tres y ocho meses, dependiendo del tipo. Entre los fármacos más frecuentemente usados encontramos a los antipsicóticos y el metilfenidato.

Encontramos más niños con TEA de sexo masculino que femenino, en una proporción de 3,8 a 1, esto coincide con lo reportado en la literatura (mayor prevalencia en varones que mujeres en una proporción de 3 a 1) ^8^, así como lo reportado por el Registro del Consejo Nacional para la Integración de la Persona con Discapacidad (CONADIS) de Perú, que reportó que al 2018 el 80,9% de los pacientes inscritos con TEA eran varones ^5^. Aún está en investigación si esto es debido a sesgos en el diagnóstico o a causas fisiológicas y sociales que permiten que las mujeres tengan mejores conductas de adaptación/compensación ^8^.

La media de edad de diagnóstico de TEA fue de 3,83 años (46 meses), cercana a la estimada a nivel global por una revisión sistemática que fue de 43,2 meses (rango: 30,9 - 74,7 meses) en niños menores de diez años ^20^. Sin embargo, la media hallada sugiere que podríamos estar ante un diagnóstico tardío con relación a otros países como Argentina o España (media de 3,30 años), que podría estar relacionado a un tiempo de espera prolongado para las atenciones médicas y psicológicas ^9^.

En los centros asistenciales de EsSalud se registran los diagnósticos usando el CIE-10. Desde el 2013 se prefiere usar al Manual Diagnóstico y Estadístico de los Trastornos Mentales Quinta Edición (DSM-5) como referente para el diagnóstico de TEA a nivel mundial ^8^. El DSM-5 para el TEA no considera categorías sino niveles de apoyo (nivel 1: requiere apoyo, nivel 2: requiere apoyo notable, nivel 3: requiere apoyo muy notable) ^7^. Registrar el nivel de apoyo de TEA correspondiente podría ser útil para diseñar programas de intervención que atiendan necesidades específicas, como las educativas.

El 21,7% fueron diagnosticados de TEA por Psicología. En el Perú, el MINSA señaló el 2022 que el diagnóstico debe ser realizado por médicos, idealmente subespecialistas pediátricos, con la contribución de otros profesionales ^21^. Las guías de práctica clínica sobre TEA suelen recomendar la participación de un equipo multidisciplinario en el proceso diagnóstico ^22^^,^^23^. Este proceso, que debe durar máximo de tres a seis meses, puede demorar hasta un año en países de altos ingresos como Canadá, por lo que se ha propuesto que profesionales de salud no médicos capacitados realicen el diagnóstico de TEA en casos poco complejos ^23^. Esta estrategia podría ser útil para ampliar la capacidad de diagnóstico de TEA en el Perú.

La distribución educativa fue similar a la reportada por el Ministerio de Educación del Perú, predominando la modalidad regular ^5^, donde menos del 10% refirió contar con apoyo del SAANEE (educación inclusiva). Un estudio realizado en Lima el 2022 encontró que existe una tendencia fuerte a excluir a los niños con TEA de instituciones de educación regular, puesto que más del 70% de los padres encuestados señalo que rechazaron a sus hijos en por lo menos un colegio, y más del 50% que habían pasado por más de un colegio ^24^. Además, hay evidencia emergente que sugiere que los adolescentes con TEA tienen menos probabilidad de recibir educación superior que sus pares coetáneos sin esta condición ^25^.

Uno de cada cuatro presentó conductas agresivas, menor a lo reportado en la literatura (35-50%) ^26^. Las conductas agresivas en niños con TEA afectan su educación, causando incumplimiento y abandono escolar, disminuyen la eficacia de las intervenciones de rehabilitación, afectan las relaciones interpersonales, provocando aislamiento social y generan estrés en los padres siendo uno de los principales motivos por los cuales buscan tratamiento farmacológico para el niño ^26^.

Más del 77% no habían completado su evaluación psicológica, posiblemente en relación con la escasez de psicólogos en el sistema de salud del Perú, sobre todo en el primer nivel de atención y fuera de la capital (Lima) ^27^, y con el hecho que el SRP-HNERM contó con solo dos psicólogas el 2022. La evaluación psicológica es fundamental en los trastornos del desarrollo infantil para describir el funcionamiento en varios dominios, particularmente en situaciones cotidianas, así como para cuantificar el nivel general de desarrollo cognitivo del niño y determinar la presencia o no de discapacidad intelectual ^28^.

La mayoría no tenían certificado de discapacidad, documento que acredita la condición de discapacidad de una persona en el Perú y permite su inscripción en el CONADIS ^22^. En el Perú, no todas las personas con TEA tienen certificado de discapacidad, sobre todo en ciudades fuera de Lima, debido a déficit de personal médico capacitado para realizarlo, dificultades para acceder a los servicios de salud, falta de conocimiento y además por ser este no obligatorio ^22^. Esta situación podría mejorar en el Perú decretando la obligatoriedad del certificado de discapacidad y creando un registro nacional de personas con TEA.

Encontramos mayores porcentajes de retraso del desarrollo en las áreas Audición y Lenguaje, Motora Fina y Personal Social. Estos hallazgos son similares a los de un estudio que evaluó el desarrollo infantil en niños menores de cinco años atendidos el 2022 en el SRP-HNERM ^19^. Así mismo, menos de la mitad recibía terapia física, posiblemente relacionado con la baja frecuencia de retraso del desarrollo en el área Motora Gruesa, en comparación con otras áreas, así como de trastornos musculoesqueléticos asociados. Esto confirma la importancia de contar en el SRP-HNERM con más terapeutas ocupacionales, terapeutas de lenguaje y especialistas en aprendizaje.

La mediana del tiempo de espera para recibir terapia ocupacional fue de ocho meses, mayor a la de terapia física y terapia de lenguaje, posiblemente porque el SRP-HNERM solo contó con dos terapeutas ocupacionales durante el 2022. Un estudio realizado el 2016 en Estados Unidos, reportó que en Tennessee los tiempos de espera de las evaluaciones e intervenciones para niños con TEA podían ser hasta de seis a doce meses ^29^.

La Organización Mundial de la Salud (OMS) publicó el 2023 el Paquete de Intervenciones de Rehabilitación para Trastornos del Desarrollo Neurológico, donde especifica las intervenciones funciones para las personas con TEA, a cargo del fisiatra, terapeutas de rehabilitación, psicólogos, entre otros profesionales de la salud involucrados, para la evaluación y el manejo de las funciones cognitivas, funciones del sueño, problemas de comportamiento, funciones de la percepción, funciones del lenguaje, el habla y la comunicación, funciones sexuales y relaciones íntimas, funciones del movimiento, funciones de la tolerancia al ejercicio, actividades de la vida diaria, relaciones e interacciones interpersonales, educación, trabajo y empleo, participación en la vida social y comunitaria, autocuidado, apoyo para el cuidador y la familia, y salud mental ^30^.

La mayoría no registraba comorbilidades asociadas. Creemos que esto podría deberse a omisión de registro, ya que el 2021 una revisión bibliográfica sistemática concluyó que las comorbilidades son altamente prevalentes en el TEA ^31^. La comorbilidad más frecuente en los niños con TEA evaluados fue el TDAH, coincidiendo con lo reportado en la literatura, donde su prevalencia en esta población puede llegar al 86% ^31^. La presencia de ambas condiciones se asocia con una mayor gravedad del TEA y un riesgo significativamente mayor de una tercera comorbilidad, especialmente ansiedad o depresión ^32^. Las comorbilidades pueden suponer un reto diagnóstico y terapéutico en niños con TEA, requiriendo enfoques de tratamiento y apoyo personalizados y adaptados ^31^.

La risperidona fue el medicamento más prescrito, cuyo uso en niños con TEA está aprobado en Estados Unidos para tratar la irritabilidad y las conductas agresivas asociadas ^33^. Una revisión sistemática y metaanálisis sobre el uso de risperidona en niños con TEA sugirió que podría ser eficaz en el tratamiento de la letargia y el habla inadecuada ^33^. Encontramos que el metilfenidato fue otro medicamento comúnmente usado, usualmente prescrito para tratar los síntomas cardinales del TDAH (inatención, impulsividad, hiperactividad) ^34^. Una revisión sistemática y metaanálisis sobre el uso del metilfenidato en niños con TEA respaldó su eficacia y tolerabilidad para el tratamiento de los síntomas del TDAH en esta población ^34^.

El presente estudio tiene algunas limitaciones que deben ser consideradas para interpretar correctamente sus resultados: 1) Al ser un estudio retrospectivo, que revisó historias clínicas electrónicas, es posible que algún dato haya sido registrado erróneamente por los médicos a cargo de las consultas en el SRP-HNERM. 2) No se pudieron recolectar otras variables relevantes por no haber estado registradas sistemáticamente en las historias clínicas electrónicas (como signos y síntomas de TEA presentes o el grado de discapacidad en la certificación). 3) El estudio fue realizado en un hospital de referencia en Lima, Perú, por lo cual los resultados podrían no ser representativos de otros establecimientos. 4) El diagnóstico de TEA fue realizado por distintos especialistas, y no tenemos información certera sobre los métodos que usaron para efectuar este diagnóstico.

Sin embargo, este es uno de los primeros estudios peruanos que ha evaluado en profundidad las características de los niños con TEA que reciben atención en Rehabilitación Pediátrica. Además, según nuestro conocimiento, este es el primer estudio en abordar este tema después de la pandemia de COVID-19 en Perú, lo que proporciona información relevante para comprender las necesidades de esta población y para hacer propuestas de mejora.

En resumen, este estudio brinda una visión profunda de las características de niños con TEA atendidos en el SRP-HNERM en Perú durante el 2022. La media de edad de diagnóstico de TEA fue mayor de tres años, y más del 75% de los pacientes no contaban con un certificado de discapacidad ni con una evaluación psicológica completa. En menos del 10% de los que recibían educación regular, esta podía calificar como inclusiva. Algunos no recibían terapias de rehabilitación y la mediana del tiempo desde la última sesión vario entre tres y ocho meses, dependiendo del tipo. Estos hallazgos resaltan la imperiosa necesidad de potenciar el diagnóstico temprano, la educación inclusiva y la evaluación y posterior certificación de la discapacidad en niños con TEA, así como de establecer intervenciones de rehabilitación más oportunas en el contexto peruano.
